# Hypopigmented Atrophic Pityriasis Versicolor: A Case of Diagnostic Dilemma

**DOI:** 10.7759/cureus.55763

**Published:** 2024-03-07

**Authors:** Sabiha Quazi, Sugat Jawade, Sudhir Singh, Khalid Khan

**Affiliations:** 1 Department of Dermatology, Datta Meghe Medical College, Datta Meghe Institute of Higher Education and Research, Nagpur, IND; 2 Department of Medicine, Datta Meghe Medical College, Datta Meghe Institute of Higher Education and Research, Nagpur, IND

**Keywords:** malassezia, perifollicular hypopigmentation, fungal infection, dermatology, pityriasis versicolor

## Abstract

Pityriasis versicolor (PV) also referred to as Peter Elam’s disease or tinea versicolor is caused by the *Malassezia* species which is a chronic-relapsing widespread mycosis. The most common sites involved are the shoulders, upper arms, back, upper trunk, and chest. Atrophying PV is a very rare variant that has rarely been reported in the Indian literature. Hence, in this case report, a 29-year-old male presented with chief complaints of multiple asymptomatic, light-colored lesions over his chest, shoulder, and arms for three months. On examination, multiple well-defined hypopigmented macules of varying sizes with fine scales were observed on the patient’s chest, shoulders, and arms. Dermoscopic examination revealed nonuniform perifollicular hypopigmentation with clearly demarcated borders, patchy scaling, and inconspicuous ridges and furrows. Moreover, a histopathological examination was performed that reported flattening of rete ridges along with fungal hyphae and spores which consequently confirmed the diagnosis. The medical intervention with antifungal agents was prescribed by the dermatologist, after which the lesion was completely resolved and the follow-up period reported no recurrence of the lesions demonstrating positive outcomes. In conclusion, diagnosing atrophic PV which is a rare variant of PV can be challenging. Hence, accurate diagnosis along with appropriate and adequate intervention can lead to the resolution of the condition and can prevent its recurrence.

## Introduction

Pityriasis versicolor (PV), which is also known as Peter Elam's disease or tinea versicolor, is a widespread mycosis that is chronic and recurrent. It is caused by a lipophilic yeast called *Malassezia *species, which is a dimorphic fungus that infects the stratum corneum leading to fungal infection. As a part of normal microbiota, numerous species of *Malassezia *fungus have been identified in the skin of healthy individuals. *Malassezia sympodialis*, *Malassezia furfur*, and* Malassezia globosa* are found to be correlated with PV [1,2].

Multiple conditions consisting of fluctuations in hormonal levels, endocrine abnormalities, hyperhidrosis, increased sebum secretions, acquired or congenital immunodeficiency, and endocrine abnormalities may facilitate the growth of the yeast and its transformation into a pathogen [1,3]. The most prevalent sites for PV are the shoulders, upper arms, back, upper trunk, and chest. Thighs and groin regions are found to be involved in rare cases. PV usually starts with oval and round, macular, coalescing, brownish-red, nonpruritic lesions, where tangential scraping may elicit distinct bran-like scaling and are present in various sizes [4,5].

PV lesions are predominantly composed of the organism's mycelial phase. As per the 1996 taxonomic revision, the genus *Malassezia *is comprised of seven distinct species, and it is a typical component of the human skin flora. The commensal yeast can change into filamentous pathogenic forms under specific circumstances. It becomes more prevalent in the late teens and peaks in the early twenties. In comparison to the temperate zones, the condition is more prevalent in tropical zones, and up to 40% of people may be infected [6]. Crowson and Magro introduced the term "atrophying PV" to describe this variant of PV in which skin atrophy rarely coexists with PV lesions [7] Hence, this case study describes a unique type of PV that is rarely reported in the Indian literature and is represented by the presence of atrophy of the patches.

## Case presentation

Patient information

A 29-year-old male presented to the dermatology outpatient department (OPD) with chief complaints of multiple asymptomatic, light-colored lesions over his chest, shoulder, and arms for three months. The patient denied any prior medical treatment to the lesions. 

Clinical examination

**Figure 1:**
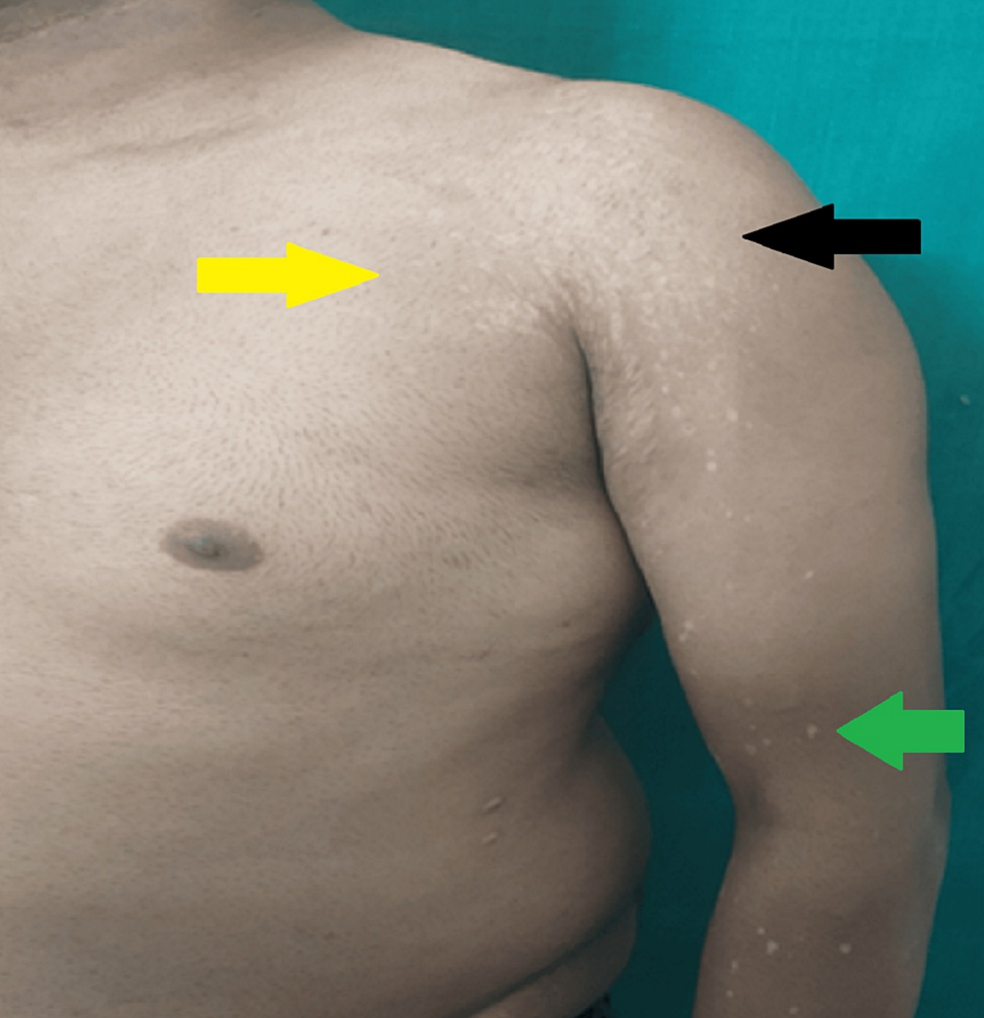
Presence of atrophic hypopigmented macules on the chest, shoulder, and arms Yellow arrow illustrates atrophic hypopigmented macules on the chest. Black arrow illustrates atrophic hypopigmented macules on the shoulder. Green arrow illustrates atrophic hypopigmented macules on the arm

On examination, well-defined multiple hypopigmented macules of varying sizes with fine scales were observed on the patient’s chest, shoulders, and arms as shown in Figure 1. On palpation, minor atrophy could be appreciated along with the depressed appearance of the patches. There were no mucosal lesions, and the systemic examination was found to be normal. A differential diagnosis of lichen sclerosus, guttate leucoderma, and atrophic PV was determined based on the above characteristics.

Diagnostic assessment

**Figure 2:**
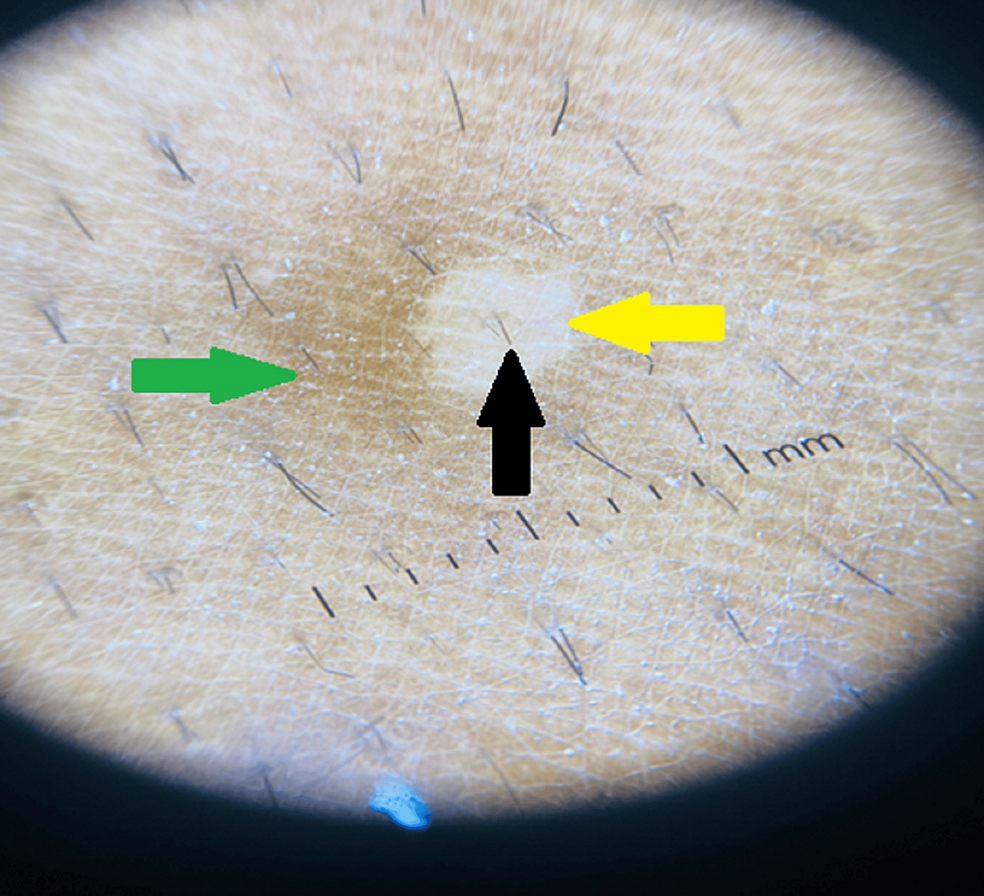
Dermoscopic examination of the lesion Black arrow illustrates hypopigmentation of the involved hair follicle. Yellow arrow illustrates the primary folliculocentric lesions showing scaling at the border along with reduced pigmentary network. Green arrow illustrates the contrast halo (hyperpigmented) ring around the primary lesion

**Figure 3:**
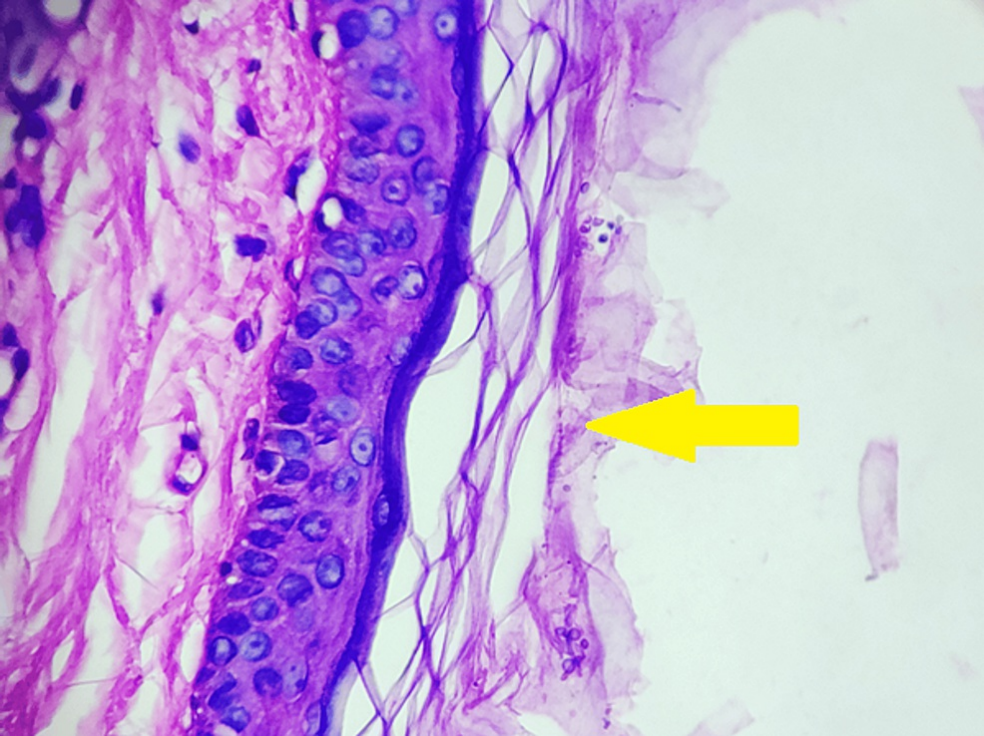
Histopathological examination of the skin lesion Hyphae are visible that are grown into strands within clumps of keratinocytes giving it the "spaghetti and meatball" appearance

Dermoscopic examination revealed nonuniform perifollicular hypopigmentation with clearly demarcated borders, patchy scaling, contrast halo (hyperpigmented) around the primary lesion, and inconspicuous ridges and furrows as illustrated in Figure 2. Moreover, from the lesion, the scraping was taken with the help of a glass slide, and a 15% potassium hydroxide (KOH) mount was prepared and examined under the microscope. The results revealed no fungal elements demonstrating a negative KOH test. Following this, the Wood’s lamp examination was performed that showed no fluorescence describing negative results. Furthermore, to confirm the diagnosis, a punch skin biopsy of 3 mm was taken and examined histopathologically that reported flattening of rete ridges under the microscope which signified a slight atrophy of the epidermis. Furthermore, the diagnosis was confirmed by microscopic examination, which showed fungal hyphae and spores that exhibited the "spaghetti and meatball" appearance which is a typical clinical presentation of atrophic PV as demonstrated in Figure 3.

Treatment

Written informed consent had been obtained from the patient before the commencement of any medical intervention. For a duration of four weeks, the patient was started with 2% topical ketoconazole application along with 100 mg oral itraconazole twice daily. Before the bath, a 15-minute contact therapy with 2.5% selenium sulfide was also provided. Following the duration of four weeks, the atrophy and skin lesions resolved, scaling was reduced, and the hypopigmented patches faded. Since atrophic PV requires a lengthier course of medical intervention, the patient was recommended to continue the intervention for a few more weeks. After three months of follow-up, there was no recurrence of the disease; however, the patient received counseling regarding the possibility of recurrence of the lesion.

## Discussion

The present case demonstrated the dermatological condition of atrophic PV which is mainly caused by the *Malassezia *species. De Graciansky and Mery originally observed skin atrophy with PV lesions in 1971 [5]. Crowson and Magro, who analyzed the clinical and histological characteristics of 12 individuals with atrophying PV, documented the pathology related to this fungal infection [7]. The pathology involved in the atrophy of the skin may be due to mechanisms such as the direct effect on nuclear factor kappa B (NF-κB) signaling by *Malassezia* or due to delayed-type hypersensitivity reactions. Additionally, two of the patients demonstrated histological evidence of degeneration of elastic fibers. This indicates that elastase released by histiocytes, which is triggered by inflammatory reactions, results in elastolysis. Moreover, the production of tumor necrosis factor α (TNF-α) and interleukin-1β (IL-1β) which are pro-inflammatory cytokines increased in response to the activation of *Malassezia *in the horny layer. Concerning these inflammatory mediators, it has been clarified that TNF-α-induced decreased proliferation and keratinocyte apoptosis lead to the atrophy of the epidermis [5,7]. Hence, this pathology can be correlated well with the findings of the present study.

However, previous investigation has indicated that prolonged application of topical steroids led to the emergence of atrophy as it prevents the production of collagen and decreases keratinocyte mitosis [5,8]. Steroid atrophy and atrophying PV differ histopathologically in that corticosteroid atrophy is related to telangiectasias, severe atrophy of the epidermis, and degeneration of the collagen framework, whereas atrophying PV is caused by elastolysis of the dermis [9]. However, in the present case, the patient reported no history of topical usage of steroids, and therefore, the lesion presented could be the result of delayed-type hypersensitivity to epicutaneous antigens derived from PV components during T helper lymphocyte release of cytokines such as TNF-α and IL-1β.

Dermoscopy, Wood's lamp examination, KOH testing, and histopathological evaluation were performed for the diagnostic evaluation in the present case study. The diagnosis of infections by dermoscopy is still in its evolving phase, although initial findings of PV through dermoscopy seem to be promising. Considering it as a supplementary tool, the assessment of PV through dermoscopy can provide beneficial diagnostic information [10]. In the present case, dermoscopic examination revealed a nonuniform perifollicular hypopigmentation with clearly demarcated borders, patchy scaling, inconspicuous ridges, and furrows and a contrast halo (hyperpigmented) ring around the primary lesion. Similar findings were observed in a study presented by Mathur et al., where the most frequent dermoscopic characteristic observed in both the hyperpigmented and hypopigmented lesions was nonuniform pigmentation within a lesion [11]. These results also aligned with the finding of Errichetti et al., who reported a white region that was fairly demarcated in PV hypopigmented lesions compared to clearly demarcated guttate vitiligo lesions [12]. Therefore, dermoscopy can help distinguish PV from other pigmentary diseases that are devoid of scales, such as pityriasis alba, vitiligo, and progressive macular hypomelanosis [13,14]. To support the findings, more extensive research comparing these findings with electron microscopy and histology is required.

In the present case, histopathological examination revealed flattening of rete ridges along with fungal hyphae and spores illustrating the "spaghetti and meatball" appearance. This presentation is considered as a classic clinical presentation of PV [1,5]. The treatment received by the patient in the present case involved 2% application of topical ketoconazole along with 100 mg oral itraconazole twice daily, and for 15 minutes with 2.5 % selenium sulfide, a short contact therapy before the bath was also given over a four-week period. Following this period, the atrophy of the skin and the lesions resolved completely, a reduction in scaling was observed, and the hypopigmented patches faded. Similar results of the intervention were observed in the previous studies [1,5,14].

## Conclusions

Atrophic PV is a very rare variant of PV and can exhibit challenges in diagnosing the condition. Investigations through dermoscopy and correlating its findings with histopathological features may aid in the accurate diagnosis of this rare variant of PV. Moreover, an appropriate and adequate medical intervention consisting of antifungal agents illustrates the complete resolution of this dermatological condition. However, long-term maintenance of treatment should be advised to prevent recurrence of the condition.
